# Impact of Ageing on Female Metabolic Flexibility: A Cross-Sectional Pilot Study in over-60 Active Women

**DOI:** 10.1186/s40798-022-00487-y

**Published:** 2022-07-30

**Authors:** Jordi Monferrer-Marín, Ainoa Roldán, Pablo Monteagudo, Iván Chulvi-Medrano, Cristina Blasco-Lafarga

**Affiliations:** 1 Sport Performance and Physical Fitness Research Group (UIRFIDE; GIUV 2013-140), University of Valencia, Valencia, Spain; 2 Physical Education and Sports Department, University of Valencia, Valencia, Spain; 3 Department of Education and Specific Didactics, Jaume I University, Castellon, Spain

**Keywords:** CHOox, Elderly, Energy expenditure, FATmax, FATox, Metabolic health, MFO, Mitochondrial dysfunction, Muscular power, Lactate

## Abstract

**Background:**

Ageing affects metabolic flexibility, although physical status could influence this relationship. This cross-sectional study aims to describe and analyse the metabolic flexibility/inflexibility in a group of active older women, together with the impact of ageing and physical status on their oxidation rates and maximal fat oxidation (MFO).

**Methods:**

Fifteen volunteers (69.00 ± 6.97 years)—from 24 women—completed an incremental cycling test until the second ventilatory threshold. Intensity increased 10 W each 3 min 15 s, starting at 30 W. Gas exchange, heart rate, rate of perceived effort, pain scale and muscle power were registered, together with lactate. VO_2_ and VCO_2_ were considered for fat and carbohydrate oxidation (FATox and CHOox; Frayn’s equation) at intensities 60%, 80% and 100% from the peak power in the test (P_100_). Psychophysiological parameters were compared at MFO/FATmax and P_100_, together with the energy expenditure calculations around MFO (included FAT and CHO contributions), and the main correlation analyses, with and without P_100_ and VO_2_ as covariates.

**Results:**

FATox was low at MFO (0.13; 95% CI [0.09–0.17] mg/min/kgFFM; 3.50; 95% CI [2.49–4.50] mg/min/kgFFM), with short oxidation-rate curves shifting down and leftward. CHOox and FATox were both low for reduced power with age (77.14 ± 18.58 W and 39.29 ± 9.17 W at P_100_ and MFO, respectively), all accompanied by a fall in energy expenditure (5.44 ± 2.58 kcal/min and 3.32 ± 1.55 kcal/min at P_100_ and MFO, respectively). Power appears as a determinant factor, given its strong and negative significant association with age (*r* = − 0.85, *p* < 0.005; *R*^2^ = 0.72) and moderate with MFO (*r* = − 0.54, *p* = 0.04; *R*^2^ = 0.29). In turn, energy expenditure shows a positive and moderate association with muscle power (*r* = 52, *p* = 0.04).

**Conclusions:**

Despite the drop in substrates oxidation with age, physical status (i.e. larger muscular power and energy expenditure) suggests a key role in the preservation of metabolic health with ageing in active women.

## Key points

- Ageing is accompanied by a shifting down and leftward of oxidation rate curves.
- Muscle power and energy expenditure, more than VO_2_ (near the second ventilatory threshold), influences MFO and CHOox_peak_ in older active women, with blood lactate production preserved and displaying a moderate association with MFO.
- Larger power at higher exercise intensities (i.e. larger energy expenditure) is associated with better fat oxidation capacity at lower loads, pointing to a shielding effect from metabolic dysfunction.

## Background

Skeletal muscle plays a key role as a paracrine and endocrine organ [1], in addition to managing glucose metabolism and mitochondrial number and function for ATP provision [2]. The loss of muscle quality, mass, and function in age-related sarcopenia processes [3] seems to have its origins in the so-called mitochondrial dysfunction, an abnormality in the physiological functions of these mitochondria [4], a hallmark of ageing [2].

The main consequence of this mitochondrial dysfunction is a limited generation of ATP by oxidative phosphorylation. Loss of muscle mass and strength go along with reduced fat oxidation (FATox), which is theoretically counteracted with greater carbohydrate oxidation (CHOox) [5]. When this phenomenon appears already in basal conditions or at very low exercise intensities, as in older adults, it is referred to as metabolic inflexibility [6, 7], a condition manifested by a low ability to switch between energy substrates in response to changing physiological conditions [8–10]. Besides age, sex [11], training level [12], nutrition status, fasting time [10] and exercise modality [6, 7] play key roles. For instance, sex variability in oxidation rates [11], especially after menopause is accompanied by a reduced mitochondrial respiratory capacity in women [13].

In this scenario, the mechanisms involved in metabolic inflexibility [14], as well as its consequences and ways of improvement, are still under investigation, especially in exercise conditions, and especially in older women where research is non-existent. Non-invasive indirect markers could be key in this assessment. To highlight, the point of maximal fat oxidation (MFO) and FATmax, the intensity at which this point is reached, important indicators of metabolism during physical exercise [15, 16], but they need more research while aging.

Therefore, the aim of this study is to describe and further understand the behaviour of metabolic flexibility/inflexibility in active older women (by means of an incremental cycling test), with the purpose of observing the impact of ageing on oxidation rates and the peak of whole-body fat oxidation produced by this population over 60. It also aims to decipher the role of the fitness level in these values. As a main hypothesis, age might impair the metabolic flexibility, reducing MFO values, being compensated by a higher CHOox, thus shifting downwards and to the left the fat oxidation curve. As a second hypothesis, unfit women will display less flexibility.

## Methods

### Participants

Twenty-four older women were recruited to participate in the study. The inclusion criteria were as follows: female over 60 years, moderately active according to the International Physical Activity Questionnaire (IPAQ) [17], and absence of any medical contraindication for physical exercise according to the physical activity readiness questionnaire (PAR-Q) [18]. The exclusion criteria were diagnosed insulin resistance, the consumption of drugs (e.g. beta blockers) that limits or conditions the practice of physical exercise, and noncompliance with any of the inclusion criteria.

Six women were discarded after the first screening, and three more failed to complete the protocol. So, Table 1 shows descriptive data of those 15 whose data were useful to determine the metabolic flexibility.

**Table 1:** Subject characteristics expressed as the mean (95% confidence intervals) and coefficient of variation (%)

Subjects (*n* = 15)	Mean (95% CI)	CV (%)
Age (years)	69.00 (65.14–72.85)	9.7
Weight (kg)	62.44 (56.61–68.26)	16.5
BMI (kg/m^2^)	25.25 (23.87–26.63)	11.1
FFM (kg)	39.83 (36.88–42.77)	13.4
Body fat (%)	35.03 (26.17–43.89)	12.7
SBP (mmHg)	133.73 (125.05–142.41)	13.2
DBP (mmHg)	78.87 (73.46–84.28)	14
SpO_2_ (%)	97.21 (96.69–97.73)	0.8
HR_baseline_	71.71 (65.00–78.43)	12.4

95% CI: 95% confidence intervals, BMI: Body Mass Index, FFM: free fat mass, SBP: systolic blood pressure, DBP: diastolic blood pressure, SpO_2_: oxygen saturation; HR_baseline_: heart rate at baseline

Women were told to refrain from strenuous exercise 24 h before the test and to follow their usual diet, maintaining their macronutrient composition and energy content, except for the pretest dinner, so as not to overly condition oxidative ration, with a meal consisting of 50% of kcal in the form of CHO. In addition, they were asked to abstain from caffeine 1.5 h and to fast at least 2 h before the test. The participants repeated the diet on both days in the study to optimize the standardization of the test [10]. Notably, they were also instructed to arrive at the laboratory well rested and were asked to travel by car or public transport.

### General Design

The current pilot study, conducted between March and July 2021, followed a single centre, cross-sectional design. After a first telephone recruitment, including questions about the medical history, physical activity and health habits, the ladies came twice to the laboratory, on two days separately from 48 h to one week.

The first day of assessment included 10 min resting seated to register heart rate (HR) with a Polar H10 band (Polar Electro Oy, Kempele, Finland), arterial oxygen saturation (SpO_2_%) through the Wristox2 3100 pulse oximeter (Nonin Medical, Plymouth, Minnesota, USA) and blood pressure (BP) by the Omron M6 sphygmomanometer (HEM-7420, Omron Healthcare, Kyoto, Japan). Then, a brief interview was conducted to ascertain the participants’ health status and the level of physical activity by means of PAR-Q and IPAQ questionnaires, followed by the determination of height (SECA 222, Hamburg, Germany) and body composition by bioimpedance (Tanita DC-430 MA S; Tokyo, Japan). Finally, there was a familiarization set with the bicycle Orbea active 700 and the smart roller Saris H3 (CycleOps Hammer Direct Drive Trainer, Saris, Madison, USA). Cadence and biomechanical adjustment to the bicycle were set for the comfort of the participant in the graded test.

On the second day, the previous healthy controls were repeated (HR, SpO_2_% and BP), followed by glycaemia baseline by means of a flash glucose monitoring system (FreeStyle Libre, Abbott Diabetes Care, Witney, UK) and baseline lactate [BLa_pre_] (Lactate Scout, SensLab GmbH, Leipzig, Germany) before the test. Then, the ladies performed the graded test on the cycle ergometer for the determination of the respiratory exchange ratio (RER), the FATmax relative to VO_2peak_ in the test (FATmax_peak_), and the VO_2_ and VCO_2_ peak values, together with the curve of FATox and CHOox during the test (see details below). The cycling protocol was chosen to guarantee safety and lower fatigue at moderate to high intensities [19], allowing the realization of the long pallets in our study.

### The Exercise Test

The graded tests to determine oxidation rates are characterized by long stages [15, 16]. The Smart Roller Saris and the Rouvy application (VirtualTraining, Vimperk, Czech Republic) allowed us to increase 10 W every 3 min 15 s, starting from 30 W to complete a minimum duration in the whole test. Fifteen sec were added in every stage due to mechanical limitations of this population [20]. Intensity was continuously monitored and adjusted with the help of an iPad tablet (Apple, Cupertino, California, USA), and the rating of perceived exertion (RPE, Borg 1–10) and visual analogue pain scale (VAS) were controlled every 1 min 30 s during the test.

The protocol aimed to reach the second ventilatory threshold (VT2), with at least two of the three following criteria: RER > 1.1, peak HR (HR_peak_) > 80% HR_max_ [21], and/or RPE > 6 [22]. Whenever a VAS > 5 and/or SpO2% < 92%, the women were invited to end the test. VO_2_ and VCO_2_ were measured by indirect calorimetry using the K4 B2 metabolic chart (Cosmed, Rome, Italy). The online gas analysers were carefully calibrated with an automated volume calibration and with a gas mixture recommended by the manufacturer prior to the start of each test. The last 60 s in each intensity were then retained to calculate whole-body fat oxidation rates [15, 16], where the substrate oxidation was calculated using Frayn’s equation, with the assumption that the urinary nitrogen excretion rate was negligible [23]:

- FATox (g/min): 1.67VO_2_ (l/min)–1.67VO_2_ (l/min)
- CHOox (g/min): 4.55VCO_2_ (l/min)–3.21VO_2_ (l/min)

Thereafter, the FATox value at MFO was calculated, considering the FFM value (mg/min/kg FFM), as it may be more appropriate when making comparisons by sex [11, 13], whereas the analysis of the CHOox ratios, since the test ended before reaching zones of maximum oxidation values of this substrate, was discarded beyond the ratio curves.

Finally, the evolution of the substrates was graphically plotted according to the intensities 60% (P_60_), 80% (P_80_) and 100% (P_100_) set from each individual peak power at the end of the test_._ The energy substrate oxidation curve was thus short, but it allowed us to analyse this heterogeneous population without leaving aside those with less physical condition.

As with VO_2_ and VCO_2_, RER, energy expenditure (EE; kcal/min)—and FATox or CHOox contributions to this, also in kcal/min—, were calculated in the last minute of each stage. Similarly to MFO, this EE markers were normalized considering the FFM (kcal/min/kgFFM). Fat and carbohydrates energy contribution was thus plotted around MFO (previous and post stages). For those women starting the test already in MFO, the two subsequent stages were considered. Their representation was based on the RER (x-axis) by means of a grouped with stacked bars graph.

### Statistics

As a first descriptive approach, the mean, standard error of the mean (SEM), confidence interval [CI 95%] and coefficient of variation (CV) were calculated, followed by graphical analysis and normality tests (Saphiro–Wilk). After the determination of MFO and the peak power in the test (P_100_), t-tests for paired samples were conducted to compare and further understand physiological parameters at these two key points of the test in our active older women. Finally, bivariate correlations for age, BLa_peak_ and FATox in MFO were performed, with and without VO_2peak_ and peak power (P_100_) as a covariate, accompanied by scatter plots and the coefficient of determination *R*^2^ to quantify the proportion of variance in one variable explained by the other in these associations, using the GraphPad Prism® 9 (Version 9.01, GraphPad Software, Inc., La Jolla, California, USA). Cohen’s *d* was calculated for the effect size, where it was considered small (*d* = 0.20–0.40), medium (*d* = 0.50–0.70) or large (*d* = 0.80–2.0) [24], while *R*^2^ was considered small (*R*^2^ = 0.04), medium (*R*^2^ = 0.25) or large (*R*^2^ = 0.64) [25].

All analyses were carried out using the Statistical Package for Social Sciences (SPSS, v. 25.0, IBM SPSS Statistics, IBM Corporation), and the significance level was set at < 0.05.

## Results

### Body Composition and Cardiorespiratory Fitness

As shown in Table 1, there was the expected heterogeneity in FFM and VO_2peak_ values. Women showed a good BMI for their age, in the lower limit of overweight [26], as well as normal-high blood pressure or prehypertensive scores according to the European society guidelines for hypertension [27]. Heart rate and saturation were appropriate for age and context, and noteworthy, the VO_2peak_ in the test, close to the oxygen uptake at VT2, was lower than similar samples in other studies because of this also lower intensity in the protocol.

### Metabolic Flexibility and Physiological Determinants

Table 2 reflects a low FATox capacity at MFO, which appeared at 79.52% of the VO_2peak_ (FATmax_peak_) and with a low increase in HR. Values such as energy expenditure (EE) (0.09 kcal/min/kgFFM) and the contribution of fat to it (0.03 kcal/min/kgFFM), seemed to be also low in this population, a contribution that could not be completely compensated by CHOox (0.06 kcal/min/kgFFM). The RER was already over 0.85, which was associated with glucose predominance [28]. In fact, CHOox was larger than FATox at this point of the test. Considering P_100_, BLa_peak_ increased significantly (*p* < 0.05) from BLa_pre_ (1.63 ± 1.03 to 7.34 ± 4.23 mmol/L). Compared to MFO, all the main psychophysiological and performance parameters (i.e. HR, RPE, VAS, and RER; and W, respectively) showed significant differences (*p* < 0.05) but not SpO_2_ (Table 2). Despite being active, power at MFO was also low.

**Table 2:** Psychophysiological parameters at maximal fat oxidation (MFO) and maximal power once over VT_2_ (P_100_)

	MFO (*n* = 14)	SEM	P_100_ (*n* = 15)	SEM
HR (bpm)	99.50 (92.17–106.83)	3.40	135.46 (125.00–145.92)*	4.88
%HR_max_	62.38 (57.36–67.40)	2.32	84.12 (77.57–90.87)*	3.03
VO_2_ (ml/kg/min)	11.77 (9.04–14.52)	1.27	16.34 (11.67–21.01)*	2.18
SpO_2_ (%)	96.85 (95.98–97.73)	0.40	95.64 (94.49–96.79)	0.49
RPE	1.5 (0.60–2.40)	0.42	5.26 (4.52–6.00)*	0.34
VAS	0.93 (0.20–1.66)	0.34	2.73 (1.16–4.30)*	0.73
EE (kcal/min)	3.55 (2.81–4.30)	0.35	5.44 (4.01–6.87)*	0.67
EE (kcal/min/kgFFM)	0.09 (0.07–0.11)	0.01	0.14 (0.10–0.17)*	0.17
FATox (kcal/min/kgFFM)	0.03 (0.02–0.04)	0.00	0.001 (0.000–0.003)	0.000
FATox (g/min)	0.13 (0.09–0.17)	0.19	0.00 (0.00–0.01)*	0.00
FATox (mg/min/kgFFM)	3.50 (2.49–4.50)	0.47	0.17 (0.00–0.40)	0.17
CHOox (kcal/min/kgFFM)	0.06 (0.04–0.08)	0.01	0.14 (0.10–0.17)	0.02
CHOox (g/min)	0.64 (0.46–0.84)	0.84	1.88 (1.35–2.41)*	0.24
CHOox (mg/min/kg FFM)	16.51 (11.62–21.41)	2.27	47.87 (34.36–61.38)	6.25
RER	0.89 (0.86–0.93)	0.17	1.19 (1.05–1.32)*	0.06
Power (W)	39.29 (33.99–44.58)	2.45	77.14 (66.42–87.87)*	4.96

Data are expressed as mean (95% CI)

SD: standard deviation; SEM: standard error of mean; 95% CI: 95% confidence interval; MFO: maximal fat oxidation; P_100%_: point of 100% of power output; HR: heart rate; %HR_max_: percentage of maximal heart rate according to Tanaka et al. [38], VO_2_: oxygen consumption; SpO_2_: oxygen saturation; RPE: rate perceived exertion; VAS: Visual Analogue Scale of pain; EE: energy expenditure; FATox: fatty acid oxidation; CHOox: carbohydrate oxidation; RER: respiratory exchange ratio; W: watts

*Different from MFO, *p* < 0.05

**Fig. 1:**
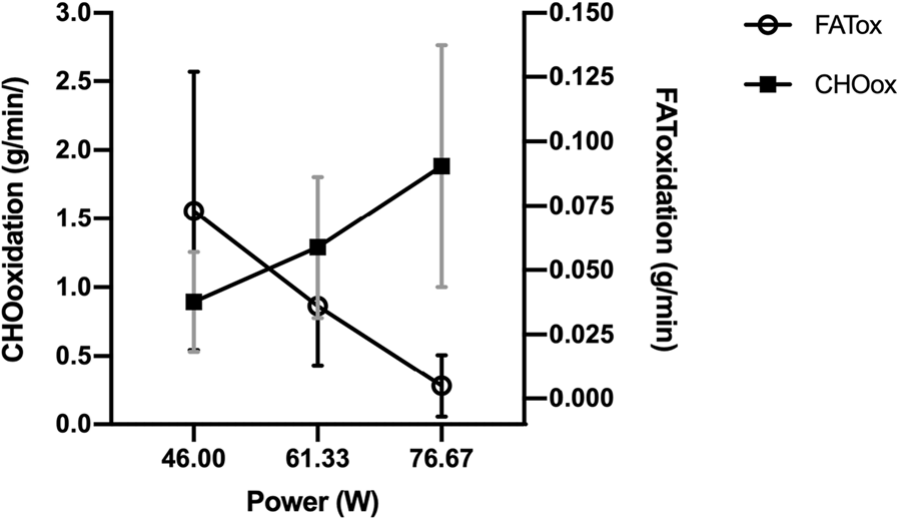
FATox and CHOox mean rates as a function of graded exercise (power). FATox: fat oxidation, CHOox carbohydrate oxidation

The three points of the ratio oxidation curve (P_60_; P_80_ and P_100_, at mean power outputs of 46.00 ± 10.80, 61.33 ± 14.40, and 76.67 ± 17.99 W, Fig. 1) reflected the limited capacity to use both FATox and CHOox, despite being active.

**Fig. 2:**
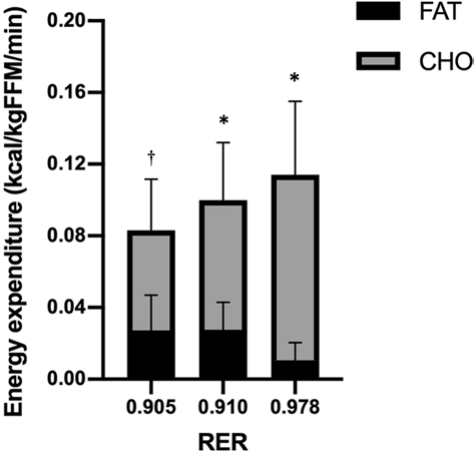
FATox and CHOox contribution to EE as a function of graded exercise (RER). *p < 0.01 in both substrates; ^†^*p* < 0.01 only in CHOox contributions. FATox: Fat Oxidation, CHOox Carbohydrate Oxidation EE: Energy Expenditure; RER: Respiratory Exchange Ratio. Values are presented as mean ± SEM

The EE evolution around MFO (Fig. 2) confirmed that EE increased due to the increase in CHOox, which was always significantly larger (*p* < 0.01). FATox dropped severely in the last stage and differences were significant only between the second and third columns (*p* < 0.01).

**Fig. 3:**
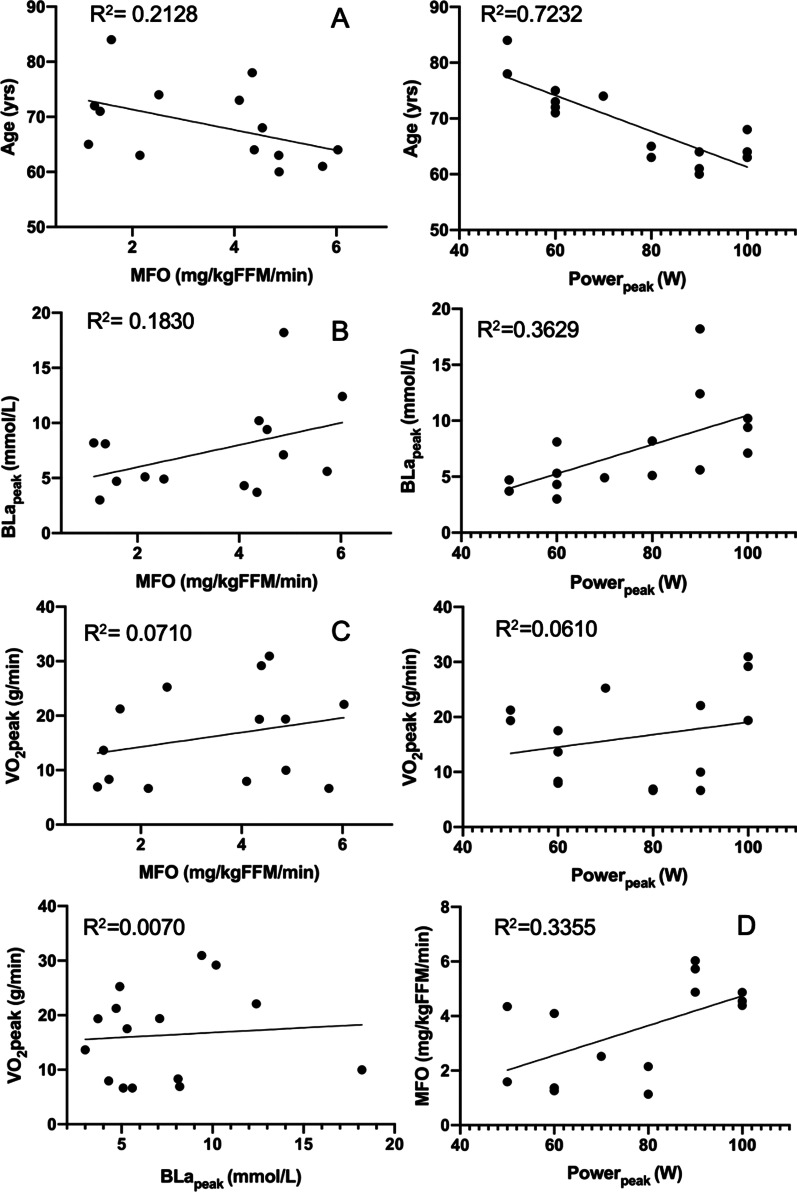
Coefficients of determination (*R*^2^) between key parameters in the test (Charts** A**–**D**: MFO associations). MFO: maximal fat oxidation, BLa_peak_: blood lactate peak, VO_2peak_: oxygen consumption at peak intensity of test

Regarding the associations (Fig. 3), an a priori analysis showed that age was large and negatively associated with P_100_ (*r* = − 0.85, *p* < 0.005) and moderate and negatively associated with BLa_peak_ (*r* = − 0.60, *p* = 0.02), while it showed no association with VO_2peak_ (*r* = 0.24, *p* = 0.38). P_100_ and VO_2peak_ appeared to be independent (*r* = 0.25, *p* = 0.37), but they were large and significant when considering age as a covariate (*r* = 0.89, *p* < 0.01).

Of utmost importance, the participants showed a unsignificant, medium size and negative correlation between age and MFO (*r* = − 0.51, *p* = 0.05), which increased to significative when this partial correlation was performed with VO_2peak_ as a control variable (*r* = − 0.59, *p* = 0.03). Instead, the MFO-age association became nonsignificant just as soon as the partial correlation was performed with P_100_ as a covariate, which was in turn large and positively associated with MFO (*r* = 0.61, *p* = 0.01). BLa_peak_ also showed a positive and moderated association with fat oxidation at MFO (*r* = 0.52, *p* = 0.04). As expected, EE displayed a medium significant association with power at P_100_ (*r* = 0.52, *p* = 0.04).

To conclude, the coefficients of determination *R*^2^ (Fig. 2) confirmed the large effect size in the association between age and P_100_ (*R*^2^ = 0.72), which decreased to medium between MFO and age (*R*^2^ = 0.21). P_100_ correlations with BLa_peak_ and MFO were also moderate (*R*^2^ = 0.36 and *R*^2^ = 0.34, respectively).

## Discussion

In line with our main hypothesis, we found an impaired value of MFO, accompanied by a shifting down and leftward of the oxidation rate curves. Both CHOox and FATox were low at an impoverished muscle power, accompanied by a reduced total EE. Aligned with our second hypothesis, preserving muscle power to reach higher intensities in exercise suggests ensuring a better ability to oxidize fat in the lower loads. In an upside-down reading, the better capacity to use both fuel sources may help to preserve muscle power. Power and EE were significantly associated between them and decreased also significantly the older the women. Notwithstanding, a greater neuromuscular capacity and a concomitant greater energetic expenditure looked responsible for a slightly higher metabolic flexibility. Since energy substrates (i.e. fat or carbohydrates) seem to follow a normal behaviour in terms of their percentage of total contribution to the EE, when compared to other populations at a similar RER, we might consider the importance of muscle power and energy expenditure when analysing metabolic flexibility/inflexibility, even in active older women.

Metabolic inflexibility with ageing has been previously described [8], confirming the specific weight of ageing in the impaired ability to combine energy substrates [29]. In fact, our MFO outcomes show a remarkable reduction in fat oxidation [0.13 (0.09–0.17 g/min)]. However, despite Amaro-Gahete et al. [30] found MFO values of 0.29 g/min and 6.78 mg/min/kgFFM, which is significantly higher than those observed in our work (0.13 g/min and 3.50 mg/min/kgFFM), his sample was ≈ 15 years younger (53.4 years compared to our mean of 69 years) and metabolic flexibility was assessed by means of a treadmill. Cycle ergometer testing means lower values because of the lower muscle mass recruited during cycling, and the association between this and the release of catecholamines during exercise [6, 7, 31], so our values might not be so aged-impaired but related to a lower performance in the test.

The energy expenditure analysis where somehow aligned with this idea and helped to better understood the older-active-women response to exercise, where energy expenditure was indeed low, but the shifting from fat to carbohydrates and their contribution to total EE was the expected accounting the RER (Fig. 2). Normalized fat-free mass (FFM) outcomes allowed the comparison with studies which also display these energy calculations and share physiological similarities like Prior et al. [32]. Comparing to Prior [32] [63 vs 69 years in our sample, FFM 48 vs 39.93 kg, and a larger gender disparity in favor of women (65%) at not very far RER intensities (0.83 vs 0.89, larger in our study)], we found only a small reduction of 0.04 kcal/min/kgFFM in the total EE (0.13 vs 0.09 kcal/min/kgFFM) in MFO point, in addition to a lower contribution of fat to this EE: 38% vs 33%; 0.05 vs 0.03 kcal/min/kgFFM. In the same line, if we compare these values with trained and young population, like in Egan et al. [33] (23.2 ± 1.6 years,69.9 ± 1.3 kgFFM), we observe a large difference in the absolute EE of almost 6.5 kcal/min lower (≈ 10 vs 3.27 kcal/min), although the contribution of fat in this EE is very similar in both populations (≈ 30% vs 33.68), all at equal intensity in terms of respiratory coefficient (0.91 ± 0.07 vs 0.91 ± 0.1).

This literature gives reason to the arguments of a not so impaired MFO but a low physical performance in older women, with a reduced or even inexistent lipolytic training zone according to triphasic model of Skinner and McLellan [34]. This idea is also supported by a recent study of our group [35]. This depleted total energy values, disadvantaged even with respect to similar populations [32], cannot be compensated, nor by fat oxidation, likely due to the lower capacity of mitochondrial oxidative enzymes (25–40%) in older people [36], nor by CHOox, possibly related to the drop in the percentage of type I fibres [37]. Both limitations explain the early cessation of exercise in the older women. The greater association of muscular power with MFO (*r* = 0.52, *p* = 0.04) and energy expenditure (*r* = 0.58, *p* = 0.03) confirms power’s responsibility in metabolic flexibility, and thus potentialities that could be attributed to the training of this neuromuscular aptitude [35].

This early fall of fat oxidation through the graded test would require the premature involvement of carbohydrates, and thus a precipitate higher RER. Of utmost importance, this phenomenon suggests being counterbalanced by training, as shown by the strong association between P_100_ and MFO in our active women, as well as the moderate influence of BLa in MFO (P_100_ *r* = 0.61, *p* = 0.02; BLa_peak_ *r* = 0.44, *p* = 0.10, respectively). Figure 3 confirms that power could explain up to 34% of the variability in MFO and up to 36% of the variability in BLa. This latter (BLa) would in turn explain 18% of the variability in MFO, which is a moderate association.

Therefore, our data indicate that muscle power is severely affected by age in older women, even being active, confirming their need for power training, both for neuromuscular and cardiovascular (i.e. metabolic) health. In addition, according to San-Millán and Brooks [10], exercise lowers circulating lactate by increasing lactate clearance, thus increasing lipid oxidation, and reducing CHOox. Those women who have preserved the ability to produce larger muscle power in our pilot study, and a proper energy expenditure pattern, have also maintained larger fat and CHOox rates, adding new reasons to increase power exercise training with age. Moreover, exercise benefits through PGC-1α participation might lead to mitochondrial biogenesis and increased lactate clearance, helping to increase the fat oxidation [10]. Muscle power becomes, thus, an important indicator from a metabolic perspective and confirms its importance in active ageing strategies.

In summary, the reduced fat and carbohydrate oxidation values because of the drop in energy expenditure in women older than 60 years, despite being active, impedes us from labelling this behaviour as metabolic inflexibility. In fact, MFO was influenced by power (peripheral factor of motor performance and women's health) and not so much by age, lactate production and VO_2_peak in the test.

To conclude, the authors acknowledge the presence of several limitations. To highlight, the lack of a control group that would allow us to talk about differences attributable to age. Besides, most of the sample were Nordic Walking practitioners, so they were not currently familiarized with the bicycle at higher intensities. This may have conditioned the end of the test; however, a first familiarization session and the VAS and RPE scales helped to ensure that women felt comfortable and secure enough to increase intensities up to VT2. On the other hand, the sample may be somehow low (due to COVID-19 pandemic limitations), which limits the analysis and conclusions that can be drawn from this research, as well as the transferability of these results. Notably, this is a pilot study, and the sample is very representative of active women over 60, since Nordic walking is a widespread sport modality at these ages. Finally, the lack of invasive procedures in the study does prevent us from outlining the mechanism behind these findings. Future studies will therefore need to explore the explanation of these phenomena using biopsies or blood samples. Moreover, a more detailed analysis of efficiency in both pathways to complement these findings, as well as further analysis of the premature and advanced glycolytic RER, could also shed light on exercise responses with ageing. In this aspect, it is worth highlighting the absence of maximum values of VO_2_, power or lactate in the protocol, as this work focused on intensities close to VT2.

## Conclusion

Contrary to expectations, energy expenditure pattern was right, but reduced, what influenced the fall in both substrates’ oxidation. Furthermore, despite the attenuation of the fitness level (i.e. muscle power) with age, it was observed that it is a relevant variable for metabolic flexibility, and therefore, a skill that needs to be worked on to prevent multifactorial declines with ageing.

Based on the findings of the present study, we believe that emphasis should be placed on increasing physical fitness in this population of older women to reverse the effects of age on metabolic flexibility, especially strength training. Given the relevance of this condition at the metabolic level, improving this key parameter could decelerate the loss of EE and increase the metabolic flexibility. This study is pioneer to investigate metabolic flexibility and shows the influence of muscle power and energy expenditure on whole-body oxidation rates in women over 60. In addition, we observed a premature RER, which could be one of the limiting peculiarities of this population.

## Data Availability

The datasets generated and/or analysed during the current study are not publicly available due to the conditions of the ethical approval provided by the Valencia University Human Research Ethics Committee. Notwithstanding, their anonymous data and analysis are available from the corresponding author on reasonable request.

## Abbreviations

BLa: Blood lactate
BMI: Body Mass Index
BP: Blood pressure
CHOox: Carbohydrate oxidation
DBP: Diastolic blood pressure
EE: Energy expenditure
FATmax: The intensity at which MFO point is reached
FATox: Fat oxidation
FFM: Free fatty mass
HR: Heart rate
IPAQ: International Physical Activity Questionnaire
MFO: Maximal fat oxidation
PAR-Q: Physical Activity Readiness Questionnaire
RER: Respiratory exchange ratio
RPE: Rate perceived effort
SBP: Systolic blood pressure
SpO_2_: Oxygen saturation
VAS: Visual analogue scale of pain
VT_2_: Ventilatory threshold II
